# Does teaching medical ethics ensure good knowledge, attitude, and reported practice? An ethical vignette-based cross-sectional survey among doctors in a tertiary teaching hospital in Nepal

**DOI:** 10.1186/s12910-021-00676-6

**Published:** 2021-08-05

**Authors:** Carmina Shrestha, Ashma Shrestha, Jasmin Joshi, Shuvechchha Karki, Sajan Acharya, Suchita Joshi

**Affiliations:** 1 Health Action and Research, Kathmandu, Nepal; 2 National Academy of Medical Sciences, Kathmandu, Nepal; 3 Nidan Hospital, Lalitpur, Nepal; 4 Nobel Medical College, Biratnagar, Nepal; 5 New York Medical College/Metropolitan Hospital Center, New York, USA; 6 Department of Neonatology, Nepal Mediciti Hospital, Lalitpur, Nepal

**Keywords:** Medical ethics, Hippocratic Oath, Medical education, Nepal, Declaration of Helsinki, Clinical ethics

## Abstract

**Background:**

Importance of awareness of medical ethics and its integration into medical curriculum has been frequently highlighted. Study 1 aimed to assess the knowledge, attitude, and reported practices of medical ethics among clinicians at Patan Academy of Health Sciences, a tertiary care teaching hospital in Nepal. Study 2 was conducted to assess whether there was a difference in knowledge, attitude, and reported practices of medical ethics among doctors who received formal medical ethics education during undergraduate studies and those who did not.

**Methods:**

Two cross-sectional surveys using self-administered questionnaires were conducted. Study 1 included 72 participants; interns, medical officers, and consultants working at Patan Academy of Health Sciences. Study 2 was a comparative study conducted among 54 medical officers who had received formal medical ethics education (Group 1) and 60 medical officers who did not (Group 2).

**Results:**

Participants who had completed post-graduate education had higher knowledge (*p* = 0.050), practice (*p* < 0.001), and overall combined scores (*p* = 0.011). Participants with ethics education had higher knowledge (*p* < 0.001), attitude (*p* = 0.001), practice (*p* < 0.001), and overall score (*p* < 0.001). Most participants preferred consulting colleagues if an ethical dilemma arose. Fewer participants had heard of the Declaration of Helsinki. Most participants thought doctors to be most capable of judging what is best for the patient (Study 1: 70.42%, Study 2 Group 1: 42.59%, Group 2: 80%). Case scenarios in which participants demonstrated poor practice were ethical issues concerning truth-telling, end-of-life decisions, treating HIV/AIDS patients, treating a minor, and reporting colleague’s errors.

**Conclusions:**

This study found that participants who have received medical ethics education have higher knowledge, attitude, and practice scores. The results further justify the need for medical ethics education to be a part of the core medical curriculum. A blame-free environment where seniors can be approached for advice should be created. Research ethics should also be given attention. During medical ethics training, ethical issues where doctors perform poorly should be given more priority and should be discussed in a country-specific context.

**Supplementary Information:**

The online version contains supplementary material available at 10.1186/s12910-021-00676-6.

## Background

Ethics is a set of standards that lays the foundation for correct behavior. It instills the concept of what is good or right and what is bad or wrong in accordance with human and social values and norms. Medical ethics is the use of these principles to guide medical care, treatment, and professional conduct [1]. Bioethics or biomedical ethics is a broad term for the study of moral issues occurring in medicine, healthcare, and biological sciences. It incorporates four major subdivisions: (1) clinical ethics, which deals with issues arising during patient care, (2) research ethics which deals with issues arising during healthcare research, (3) professional ethics, which deals with professional conduct, duties, and responsibilities of healthcare professionals, (4) public policy ethics which deals with the formulation of laws regulating bioethical issues [2]. Medical ethics falls under the professional ethics subdivision of bioethics and deals with conduct in the medical profession and dilemmas arising during medical practice. Terms such as medical ethics and clinical ethics are often used interchangeably, though there are subtle differences. Clinical ethics, in particular, involves decision-making during clinical practice with the primary goal of improving the quality of patient care [3]. Additionally, the code of ethics is a fundamental guide that regulates exercises of qualified professions. It includes the Hippocratic Oath and code of ethics compiled by the governing medical authority of the particular country [2, 4].

History suggests the practice of ethics in medicine since ancient times, documented as Charaka Samhita during the seventh century BC [5]. With advances in modern medicine starting from the oath of Hippocrates, the code of medical ethics has been revised over time as the Declaration of Geneva, which is read along with the World Medical Association (WMA) International Code of Medical Ethics [6, 7]. While these codes guide professional physician conduct, various methods (Nijmegen method, Dilemma method, Padova method, Four Quadrant Approach, and several others) have been used for ethical case deliberation to approach complex cases with difficult treatment and care decisions [8, 9]. Furthermore, several codes pertaining to research ethics have been developed over time, like the Nuremberg Code, the Declaration of Helsinki, the Belmont Report, the Emanuel framework, and others [6, 7]. Apart from these, different physician associations have proposed several statements like the Declaration of Tokyo, the Declaration of Hawaii, the Declaration of Malta, and the Regulations in Time of Armed Conflict, which governs the medical ethical position of the physician in specific issues and situations [10]. In addition, the four fundamental principles of medical ethics proposed by Beauchamp and Childress in 1979—autonomy, justice, beneficence, and non-maleficence have been considered the standard framework to analyze ethical situations arising during the practice of medicine [4, 11].

Besides clinical skills, decisions regarding health care also require ethical expertise. Moreover, problems like doctor–patient relationship breakdown, increasing litigations, rapid changes in medical practice with progress in science and technology, and increasing demand for responsible healthcare stresses the importance of proper medical ethics education [12, 13].

Medical ethics education is an attempt to foster social values and interpersonal skills to help practice medicine. It teaches doctors about the role of values in their relationship with the patients, fellow doctors, and society [14]. This education targets to make a morally sound physician with the necessary knowledge, skills, and attitudes to become a competent practitioner [6]. The WMA recommends the mandatory inclusion of medical ethics education in the undergraduate curriculum of medicine with an adequate number of skilled faculty members [15]. Furthermore, teaching medical ethics from the early years of basic science and continuing through clinical years can be more contextual and effective [12]. Diversity can be observed in the content of the education material, faculty skillset, teaching and assessment methods in different countries, and various institutions within the same county [14, 16]. We must consider country-specific culture and laws to develop a more relevant ethics curriculum in a given context.

### Medical ethics curriculum in Nepal

Medical schools in Nepal are governed by either of the two universities, Tribhuvan University (T.U.) or Kathmandu University (K.U.). Apart from them, there are two autonomous institutions, B.P. Koirala Institute of Health Sciences (BPKIHS) and Patan Academy of Health Sciences (PAHS). A World Health Organization (WHO) report stated that ethical teaching in medical schools of the South-East Asia region, taught chiefly as part of forensic medicine, is deemed highly inadequate [17]. Nepal Medical Council (NMC), which is responsible for the development and revisions of the medical curriculum, has subsequently revised the curriculum and currently recommends the model curriculum of medical ethics developed by WHO, which includes at least 15 hours of theory classes and six hours for discussion [18, 19].

The T.U. of Nepal revised its curriculum in 2009, incorporating the new medical ethics teaching, and directed to be followed in all institutions under T.U. [20]. However, the implementation process has been sluggish and yet to be started in medical colleges under T.U. Kathmandu University also has included medical ethics in its curriculum. However, the department responsible for teaching the subject has not been clearly defined [21]. The curriculum of BPKIHS has a small section of medical law and ethics under the forensic medicine curriculum, and the objectives are not in accordance with the recommended ethics curriculum [22].

### Medical ethics curriculum at PAHS

PAHS is a major tertiary teaching hospital located in Kathmandu, Nepal. PAHS was established as a teaching hospital in 2008 A.D. with the intake of students starting from 2010 A.D. PAHS has incorporated medical ethics in the undergraduate Bachelor of Medicine, Bachelor of Surgery (MBBS) curriculum since its establishment [23]. The specific objectives include making the students recognize and discuss key ethical issues in medicine and discuss legal solutions and ethical values in everyday and controversial medical situations. The teaching–learning methods to achieve these objectives are frequent lectures, Problem Based Learning (PBL) sessions, and ethical case presentations and discussions throughout the five-and- a-half-year undergraduate course [23].

Medical ethics is introduced to students during the first two months of medical school (introductory block) under the subject Introduction to Clinical Medicine (ICM) [24]. During the first two years of the basic science course, medical ethics is incorporated in frequent lectures on topics ranging from professionalism, physician charter, core principles of ethics, duty of care, end of life decisions, human rights, and allocation of resources. Multiple PBL cases with components of ethical principles and one entirely ethics-based PBL session lasting a week are included to encourage the students to reflect on ethical issues and promote self-directed learning [24]. During three years of clinical sciences, students are mandated to maintain a learning log of at least one ethically challenging case per rotation (every eight weeks) in different clinical departments. Students are required to present at least one ethical case in each year of clinical science which is supervised by the attending physician, attended by classmates, and includes extensive post-presentation discussion. The students are also assessed on their knowledge regarding medical ethics during exams via Multiple Choice Questions (MCQs) and Problem Based Questions (PBQs) [24]. During Community Based Learning and Education (CBLE), students are posted at various levels of the health system of Nepal, ranging from working with female community health volunteers to working at a district hospital. One of the specific objectives of CBLE is to describe health care ethics and explore the dilemma of health care ethics in the community. The students are required to submit a learning log on an ethically challenging case faced during such rural postings and maintain a daily diary including such experiences [24, 25].

### Study 1

The WHO module for teaching medical ethics to undergraduates states the role of clinical teachers as role models and how ethical practices in a clinical setting can be mostly learned through observation and imitation [6]. Ensuring good knowledge, attitude, and reported practice of medical ethics among practicing physicians would help them act as positive role models to undergraduate medical students. Study 1 was conducted with the aim to assess the knowledge, attitude, and reported practices (KAP) of medical ethics among clinicians practicing at PAHS.

## Methods

### Study design, setting, and participants

This is a cross-sectional study conducted in PAHS. At the time of this study, the first batch of PAHS had not yet started the internship year. The study was carried out from August 2015 to September 2015 among interns, medical officers, and consultants working in six major departments of PAHS: surgery, medicine, pediatrics, orthopedics, gynecology/obstetrics, and emergency. These six departments were selected purposively because, as per curriculum, medical students spend a significant portion of their undergraduate studies in these departments [24].

In this study, interns are medical graduates who have completed the MBBS course and are currently undertaking one year of mandatory internship, which includes general clinical training in different departments before practicing independently [26]. Medical officers are graduates who have completed their internships and can practice independently. Consultants are doctors who have completed postgraduate education and have specialization in a particular medical specialty. At the time of this study, PAHS did not have a postgraduate program.

### Development of survey instrument

A self-administered questionnaire in English was used (see Additional file 2). The questionnaire was developed through multiple extensive discussions among the investigators and a focus group of four national and international experts consisting of the head of the department of pediatrics, assistant professor of the pediatrics department, professor of the department of general practice and emergency, and an international visiting faculty at the department of general practice and emergency at PAHS.

### Content of questionnaire

The tool consisted of four parts. Part A included questions to record sociodemographic variables—age, gender, qualification, current designation, department of practice, years of work experience, institute, and the country of undergraduate and postgraduate study, as appropriate.

Part B of the questionnaire was adapted from a study in Barbados conducted by Hariharan et al. with permission [27]. Questions included frequency of ethical problems encountered in practice, preference for consultation on an ethical issue should it arise, source of knowledge of ethics, views on the importance of ethics, rating of knowledge regarding medical ethics, and codes of ethics. Questions were asked whether respondents knew about the presence or absence of a clinical ethics committee and legal advisor at PAHS and who they thought was the most capable of judging what is best for the patient.

Part C included ten scenario-based questions. The ten scenarios contained ethical issues framed in a medical context and involved medical ethical principles. We developed the cases based on real-life experiences shared by the medical students during ‘ethical presentations’ as a part of assessment during Clinical Year I and the experiences shared by teachers. We consulted various medical ethics textbooks in the preparation of the vignettes [28–30]. The ten cases were based on commonly encountered ethical issues during clinical practice in Nepal. The WMA Ethics Manual [2] divides the ethical topics to be learned in medical school into four broad domains: physicians and patients, physicians and society, physicians and colleagues, and medical research. Under physicians and patient domain, there were eight cases on the following ethical topics—one case each on informed consent (Case 1), truth-telling/disclosure (Case 2), confidentiality (Case 3), treating minors (Case 4), contraception (Case 9) and three cases on end-of-life decisions concerning Do Not Resuscitate (DNR), euthanasia and withdrawal of treatment (Case 5, 6, 7). Under the Physician and Society domain, there was one case (Case 8) on ethics related to reportable illness (HIV/AIDS). Under the Physician and Colleague domain, there was one case (Case 10) on ethics related to physician and colleague relationships. There were no cases related to medical research.

KAP was assessed in each of the ten cases. The reported practice was evaluated with a multiple-choice question asking what the participant would have done if they were in the doctor’s position. Four different options were provided, and the best option was given a score of one. Although ethical decisions rarely fall in the discrete dichotomy of right or wrong, teaching ethical practice to undergraduate medical students aims to instill the best practice. In total, there were ten questions to assess practice.

Attitude towards actions related to ethical issues was assessed by asking the participant how strongly they felt whether the doctor’s action in each case scenario was ethical using a four-point Likert scale. Depending on the case and ethical issue involved, the participants’ answer was dichotomized to right or wrong, and each right attitude was given a score of one. In total, there were ten questions to assess attitude.

Knowledge in each case scenario was assessed using two sets of questions (1) Has any principle of ethics been breached? (Yes/No) and (2) Which is the main principle of ethics involved? The four core principles—autonomy, non-maleficence, beneficence, and justice were used in the measure of the second question, along with confidentiality which is embedded within the principle of autonomy. Case 8, involving ethical issues arising while treating an HIV/AIDS patient, had two additional questions apart from the two mentioned above (1) Has any principle of ethics been followed? (Yes/No) (2) If yes, what is the main principle that has been followed. In total, there were 22 questions to assess knowledge. The operational definitions of the main principles of ethics used in the study were as follows [2]. Autonomy is defined as patients have the right to determine their own healthcare unless deemed incompetent. Justice is the fair distribution of benefits and burdens of care across society. Beneficence is doing good for the patient and society. Non-maleficence is ensuring no harm is being done to the patient or society.

Part D was a short questionnaire regarding the respondents’ reflections after going through various case scenarios. Questions were asked to assess whether participants rated their knowledge related to clinical ethics and the relevancy of clinical ethics in work practice differently after having gone through the clinical vignettes. This was a self-administered questionnaire, so respondents were also asked whether they had used any resources to answer the questionnaire.

### Validity and reliability of questionnaire

Members of the focus group evaluated the face and content validity of the questionnaire. A pre-test was conducted among 12 randomly selected doctors; two doctors from each of the six chosen departments. We obtained constructive feedback on the readability of the questionnaire from each of the pre-test participants. The feedback was incorporated in our final questionnaire tool. The internal consistency reliability of the pre-test results across the items in the questionnaire was calculated as Cronbach’s alpha 0.715 for the primary outcome, knowledge. However, Cronbach’s alpha for practice and attitude question items was below 0.7. Deleting any item in the scale did not alter the score. We proceeded with the questionnaire despite the low Cronbach’s alpha score taking into account content and face validation of the tool and small pre-test sample size.

### Sample size and sampling procedure

At the time of the study, there were 174 doctors employed at PAHS, including interns, medical officers, and consultants. The question assessing knowledge regarding the main principle involved was considered the primary outcome of the study. The proportion of pre-test participants answering this question correctly was 51.5%. The sample size for the study was calculated using a sample size for a finite population with the pre-test proportion. The minimum required sample size was calculated to be 63 with a 10% margin of error and 95% confidence level. However, considering a 15% incomplete response rate, the sample size was increased to 72. The pre-test participants and focus group members were excluded during the sampling. Stratified simple random sampling was used, and respondents were selected by lottery method. Participants were stratified based on the selected clinical departments, and the number of participants from each was calculated based on probability proportional to size.

### Ethical consideration

This study was approved by the Institutional Review Committee of PAHS, Nepal, with the Reference No. std1508031078. All participants were informed about the study’s objective, the voluntary nature of participation, and the right to refuse participation at any time. We obtained written informed consent before the administration of the questionnaire with the participant’s signature. Confidentiality was ensured with the use of identification codes, and no personal identifiers were taken.

### Data collection and analysis

Data were collected by administering a paper-based questionnaire to individual selected participants. All participants returned the questionnaire. Data was entered in Epi-info 7 with unique codes to each respondent, and analysis was performed with IBM SPSS Statistics version 20 (IBM, Armonk, NY, USA). The pairwise deletion was carried out for missing data where missing observations were ignored, and analysis was done on variables present. The number of observations analyzed is mentioned in the tables next to each category.

Part A consisting of sociodemographic factors, and Part B consisting of general questions related to medical ethics, were presented as frequency and percentages. The normality of distribution of the KAP score and subscales scores were examined with the Shapiro–Wilk normality test. Means and standard deviations or median and interquartile range of the scores were also reported depending on whether the data followed a normal distribution. Independent sample T-test for parametric data and Mann–Whitney test for non-parametric data was used to examine the association between KAP scores, subscales and qualification, country of MBBS, and use of resources. ANOVA was used for parametric data and Kruskal–Wallis for non-parametric data to compare KAP scores, subscales against different designations and departments. A *p* value of less than or equal to 0.05 was considered as the level of significance. Spearman’s Test was used to check for correlation between years of clinical practice and KAP scores and subscales.

## Results

A total of 72 doctors working at PAHS were included in the study with a 100% response rate. The sociodemographic characteristics of the participants are summarized in Table 1. The mean age of the participants was 29.70 ± 6.26 years, and 65.71% (46) were male. The mean duration of clinical practice was 4.00 ± 5.55 years, and 65.28% (47) of the participants had completed undergraduate studies (Table 1).

**Table 1:** Demographics characteristics of respondents in study 1

Characteristics	Category	n (%)
Age (years) (N = 71)	20–25	14 (19.72)
	26–30	33 (46.48)
	31–35	17 (23.94)
	≥ 36	7 (9.86)
Sex (N = 70)	Male	46 (65.71)
	Female	24 (34.29)
Qualification (N = 72)	MBBS	47 (65.28)
	MD/MS	25 (34.72)
Designation (N = 72)	Interns	11 (15.28)
	Medical officers	36 (50)
	Consultants	25 (34.72)
Department (N = 72)	Emergency	17 (23.61)
	Gynaecology/obstetrics	14 (19.44)
	Medicine	16 (22.22)
	Orthopedics	5 (6.94)
	Pediatrics	11 (15.28)
	Surgery	9 (12.5)
MBBS completed from (N = 70)	Nepal	26 (37.14)
	Abroad	44 (62.86)
Years of practice after MBBS (N = 68)	Less than 1 years	23 (33.82)
	Less than 5 years	26 (38.24)
	Less than 10 years	10 (14.71)
	More than 10 years	9 (13.24)

Due to missing data, not all category groups sum to 72

*N* number of participants

### General view on ethics

If an ethical dilemma arose, 54.90% (39) participants would prefer to consult their colleagues first, followed by the head of the department (32, 45.10%). More than half of the participants cited work experience as the primary source of knowledge regarding medical ethics (46, 63.89%) (Table 2). Participants rated the relevance of medical ethics in work practice high (median 8 ± IQR 3, on a scale from ‘1’ low to ‘10’ high). Most respondents faced ethical issues at least once a month (35, 48.60%), while 22.20% (16) claimed to have not encountered any to date (see Additional file 1). All respondents agreed that medical ethics should be a part of medical education.

**Table 2:** Source of knowledge of medical ethics for study 1 (N = 72)

Source	n (%)
Work experience	46 (63.89)
Lectures during MBBS	44 (61.11)
Books/literature	31 (43.05)
Seminar/workshops/CME	19 (26.39)
Lectures during PG	11 (15.27)

Most respondents had heard of the Hippocratic Oath (66, 92.96%) and cited having good knowledge regarding it (median 7 ± IQR 3, on a scale from ‘1’ low to ‘10’ high). More than half of the respondents were aware of the Nepal Medical Council code of ethics (54, 76.06%) and responded to have good knowledge (median 6 ± IQR 3). In comparison, only 46.48% (33) respondents had heard of the Declaration of Helsinki, and knowledge regarding it was rated low (median 0 ± IQR 4). There was no clinical ethics committee present in Patan Hospital at the time of the study. However, 52.24% (35) of the respondents were unaware of it but rated the importance of a clinical ethics committee in a hospital high (median 9 ± 3) (see Additional file 1). Nevertheless, 58 (85.30%) knew about the presence of a legal advisor in Patan Hospital.

When asked who they thought was the most capable of judging what is good for the patient, only 29.58% (21) of the respondents considered the patients themselves to be the best judge, while 70.42% (50) thought that doctors are the most capable (see Additional file 1). Few participants (13, 18.60%) claimed to have used resources to complete the questionnaire. However, no significant difference (*p* = 0.990) was observed in the knowledge score between those who used resources and those who did not.

### Assessment of knowledge, attitude, and reported practice

There was a positive but weak to moderate correlation between years of clinical practice with attitude (r = 0.262, *p* = 0.034), reported practice (r = 0.476, *p* < 0.001), and overall KAP score of medical ethics (r = 0.313, *p* = 0.016) (see Additional file 1).

Doctors who had completed postgraduate education had statistically significant and higher knowledge scores (*p* = 0.050), practice scores (*p* < 0.001), and overall combined KAP scores (*p* = 0.011). However, no significant difference was observed in attitude scores (*p* = 0.061) among those who had completed postgraduate and those who had only completed undergraduate studies (Table 3). Similar results were also observed where consultants had higher practice (*p* = 0.003) than medical officers and interns. However, there was no significant difference in knowledge score (*p* = 0.144), attitude (*p* = 0.158), and combined KAP score (*p* = 0.063) among the three groups of participants (Table 3).

**Table 3:** Assessment of knowledge, practice, attitude and combined KAP of medical ethics for study 1

	Knowledge			Attitude			Practice			Combined KAP		
	N	Mean ± SD (n = 22 questions)	*p* value	N	Median ± IQR (n = 10 questions)	*p* value	N	Median ± IQR (n = 10 questions)	*p* value	N	Measure of Central tendency ± dispersion (n = 42 questions)	*p* value
*Qualification*												
MBBS	43	12.00 ± 2.96	0.050^c^	46	7.00 ± 2.00	0.061^f^	47	6.00 ± 3.00^b^	0.001^f^	42	24.52 ± 4.20^a^	0.011^c^
MD/MS	21	13.67 ± 3.45		24	8.00 ± 2.50		24	7.00 ± 2.00^b^		19	28.00 ± 5.84^a^	
*Designation*												
Intern	10	11.80 ± 3.19	0.144^d^	11	7.00 ± 2.00	0.158^e^	11	6.00 ± 4.00^b^	0.003^e^	10	24.50 ± 10.00^b^	0.063^e^
Medical Officer	33	12.06 ± 2.94		35	7.00 ± 1.00		36	6.00 ± 3.00^b^		32	24.00 ± 4.00^b^	
Consultant	21	13.67 ± 3.45		24	8.00 ± 2.50		24	7.00 ± 2.00^b^		19	27.00 ± 6.00^b^	
*Department*												
Emergency	17	11.94 ± 3.34	0.093^d^	15	7.00 ± 2.00	0.818^e^	17	6.00 ± 1.50^b^	0.899^e^	15	25.93 ± 4.92^a^	0.477^d^
Gynae/Obs	12	11.58 ± 3.58		14	7.00 ± 1.25		13	6.00 ± 3.00^b^		11	23.73 ± 5.71^a^	
Medicine	14	11.79 ± 2.01		16	7.00 ± 2.00		16	6.00 ± 1.00^b^		14	24.86 ± 3.48^a^	
Orthopedics	3	13.00 ± 2.00		5	7.00 ± 1.50		5	7.00 ± 4.50^b^		3	24.67 ± 3.06^a^	
Pediatrics	11	15.00 ± 3.07		11	7.00 ± 1.00		11	4.00 ± 4.00^b^		11	28.00 ± 5.70^a^	
Surgery	7	13.14 ± 3.63		9	6.00 ± 1.50		9	6.00 ± 3.50^b^		7	26.00 ± 5.97^a^	
*MBBS completed from*												
Nepal	24	12.92 ± 3.82	0.539^c^	25	7.00 ± 2.00	0.650^f^	25	6.00 ± 2.00^b^	0.122^f^	22	26.00 ± 8.25^b^	0.261^f^
Abroad	38	12.39 ± 2.81		43	7.00 ± 2.00		44	6.00 ± 3.00^b^		37	25.00 ± 7.50^b^	

Due to missing data, not all category groups sum to 72

*N* number of participants

^a^Mean ± SD

^b^Median ± IQR

^c^Independent T-test

^d^ANOVA

^e^Kruskal–Wallis

^f^Mann–Whitney U

No significant difference was observed in the knowledge (*p* = 0.093), practice (*p* = 0.899), attitude (*p* = 0.818) and combined KAP score (*p* = 0.477) across doctors of different departments. There was no difference in knowledge (*p* = 0.539), practice (*p* = 0.122), attitude (*p* = 0.650) and combined KAP score (*p* = 0.261) between doctors completing their MBBS studies in Nepal or abroad (Table 3).

In 8 out of 10 ethical case scenarios, more than half of the participants could identify if there had been a breach of ethics. However, less than half of the participants were able to identify the main principle involved in each case scenario in most cases (Table 4). Cases in which half of the participants did not choose the best practice was ethical issues related to truth-telling (Case 2), end-of-life decisions related to euthanasia (Case 6), treating HIV/AIDS patients (Case 8), and physician and colleague relationship related to reporting colleague’s error (Case 10) (Table 4, see Additional file 1).

**Table 4:** Performance of study participants in each case scenario in study 1

Case	Ethical issues related to	N	Knowledge of presence of breach of ethics n (%)	N	Knowledge on principle of ethics involved n (%)	N	Attitude n (%)	N	Practice n (%)
1	Informed consent	72	64 (88.89)	71	54 (75.00)	71	68 (94.44)	72	69 (95.83)
2	Truth-telling	72	64 (88.89)	70	24 (33.33)	72	63 (87.50)	72	22 (30.56)
3	Confidentiality	71	50 (69.44)	72	57 (79.17)	72	59 (81.94)	72	61 (84.72)
4	Treating minors	72	32 (44.44)	71	23 (31.94)	72	36 (50.00)	72	37 (51.39)
5	End-of-life decisions (DNR)	72	37 (51.39)	71	32 (45.07)	72	36 (50.00)	72	49 (68.06)
6	End-of-life decisions (euthanasia)	72	53 (73.61)	71	32 (44.44)	72	64 (88.89)	72	35 (48.61)
7	End-of-life decisions (withdrawal of treatment)	72	44 (61.11)	71	33 (45.83)	72	41 (56.94)	71	55 (76.38)
8 Part 1	Reportable illness (HIV/AIDS)	71	38 (52.78)	69	31 (43.05)	72	50 (69.44)	72	34 (47.20)
8 Part 2	Reportable illness (HIV/AIDS)	71	57 (79.16)	70	26 (36.11)	-	-	-	-
9	Contraception	72	29 (40.28)	71	40 (55.56)	72	31 (43.06)	72	40 (55.60)
10	Physician and colleague relationship (reporting error)	72	52 (72.22)	71	29 (40.28)	71	59 (81.94)	72	28 (38.90)

*N* number of participants

Due to missing data, not all category groups sum to 72

### Study 2

PAHS has integrated medical ethics into its curriculum through frequent lectures, problem-based learning sessions, ethical case presentations, and discussions throughout the five-and-a-half year undergraduate course. The students are also assessed on their knowledge regarding medical ethics during exams. However, there is no available evidence of whether teaching ethics at the undergraduate level improves ethical practice among physicians or not. Study 2 was done to assess if there is a difference between knowledge, attitude, and reported practice of medical ethics among doctors who were taught formal medical ethics courses during undergraduate level and those who were not.

## Methods

### Study design, setting, and participants

This is a comparative cross-sectional study conducted among two groups of medical officers. Group 1 consisted of doctors who had recently graduated from PAHS in 2016 and graduates from other medical schools who had received adequate formal medical ethics education during MBBS and worked as medical officers at PAHS. Group 2 consisted of graduates from other medical schools who had not received formal medical ethics education during MBBS and worked as medical officers at PAHS. Our pre-study assumption was that only PAHS graduates of 2016 had received formal ethics education; however, during our study, graduates from Kathmandu University School of Medical Sciences (KUSMS) and KIST Medical College also reported receiving intensive medical ethics education besides those covered in forensic medicine. Therefore, we included graduates from PAHS, KUSMS, and KIST medical college in Group 1. The study was conducted from March 2017 to April 2017.

### Sample size and sampling procedure

At the time of the study, there were 54 graduates in the first batch of PAHS, 14 of whom worked as medical officers at PAHS itself. There were 95 other medical officers working at PAHS who were graduates from different medical schools. The sample size for Study 2 was calculated using two-arm sampling using the mean KAP score of participants who had completed MBBS in Study 1. The minimum sample size was calculated to be 50 in each group with 80% power for both groups to have a mean KAP score difference of five at a 10% level of significance. However, considering incomplete response rates, the sample size was increased to 60 for each group. Convenience sampling was done where the questionnaire for the study was distributed through emails to all graduates of PAHS who graduated in the year 2016 and to all medical officers currently employed at PAHS.

### Data collection and analysis

The same questionnaire tool used in Study 1 was used in this study with some adaptations. Questions pertaining to postgraduate education were removed from Part A of the questionnaire. Also, five additional questions related to the teaching of medical ethics during MBBS were asked (see Additional file 3).

The questionnaire was created in Google Form. The data collection was done online by emailing the web-based form link to all eligible participants. The first page of the form contained the informed consent. Participants willing to participate could proceed to the questions by clicking “Next.” Participants were asked to fill the questionnaire only once to prevent duplicate entries. Participants could review and change their answers only before submitting the form. Data collection was stopped once the desired sample size for each group was reached. The confidentiality of the participants was assured by the use of unique codes.

SPSS version 20 was used for the analysis of the data. Part A and Part B were presented as frequency and percentages. The normality of distribution of the KAP score and subscales scores were examined with the Shapiro–Wilk normality test. Chi-square analysis was used to evaluate the statistical significance of differences in age and country from where MBBS was completed, while the Mann–Whitney test was used to evaluate differences in age and months of clinical practice among the two study groups. Independent T-test for parametric data and Mann–Whitney test for non-parametric data were used to compare KAP scores and subscales between the two groups. Listwise deletion was done for missing data, where participants with incomplete responses were excluded from the study. A total of 120 forms were received; however, 6 (5%) were excluded due to incomplete responses.

## Results

A total of 114 medical officers currently practicing at PAHS were included in our study. Among them, Group 1 (54, 47.36%) consisted of respondents who had medical ethics lectures during their MBBS program, and Group 2 (60, 52.63%) consisted of respondents who did not have medical ethics lectures. Among 54 doctors in Group 1, the majority were graduates of PAHS (37, 68.51%).

Table 5 describes the sociodemographic characteristics of participants included in Study 2. In both the groups, the majority of respondents were male (Group 1: 35, 64.81% and Group 2: 32, 53.33%). The mean age of participants in Group 1 was 25.80 ± 1.41 years and in Group 2 was 25.72 ± 1.38 years (Table 5). No significant difference was observed in age (*p* = 0.913) and gender (*p* = 0.214) among participants in Group 1 and Group 2. All participants from Group 1 had completed their MBBS from medical schools in Nepal (54, 100%), whereas 63.33% (38) participants from Group 2 had studied MBBS abroad (Table 5). The mean duration of clinical practice following completion of MBBS in Group 1 was 5.57 ± 3.08 months, while that in Group 2 was 10.12 ± 8.90 months. Statistically significant differences were observed between the two groups regarding the country of MBBS completion (*p* < 0.001) and duration of clinical practice (*p* < 0.001) (Table 5).

**Table 5:** Demographics characteristics of respondents in study 2

Characteristics	Category	Group 1 (n = 54)	Group 2 (n = 60)	*p* value
		n (%)	n (%)	
Age (years)	20–25	25 (46.30)	29 (48.33)	0.913^a^
	26–30	29 (53.7)	30 (50.00)	
	31–35	0 (0)	1 (1.67)	
Sex	Male	35 (64.81)	32 (53.33)	0.214^b^
	Female	19 (35.19)	28 (46.67)	
MBBS completed from	Nepal	54 (100.00)	22 (36.67)	< 0.001^b^
	Abroad	0 (0)	38 (63.33)	
Months of practice after MBBS	< 5	38 (70.37)	17 (28.33)	< 0.001^a^
	6–11	13 (24.07)	26 (43.34)	
	> 12	3 (5.56)	17 (28.33)	

*n* Number of participants

^a^Mann–Whitney

^b^Chi-square test

### General view on ethics

Among the two groups, 72.22% (39) participants in Group 1 and 50% (30) participants in Group 2 preferred to consult with their colleague if an ethical dilemma arose, followed by the head of the department by 20.37% (11) participants in Group 1 and 38.33% (23) in Group 2 (see Additional file 1). The source of knowledge of medical ethics among participants in both groups was mainly from lectures during the MBBS study (Group 1: 92.59%, 50 and Group 2: 70%, 42) followed by work experience (see Additional file 1). Although there was no formal medical ethics education during undergraduate education in Group 2, a major source of knowledge was few lectures in forensic studies during medical school.

**Fig. 1:**
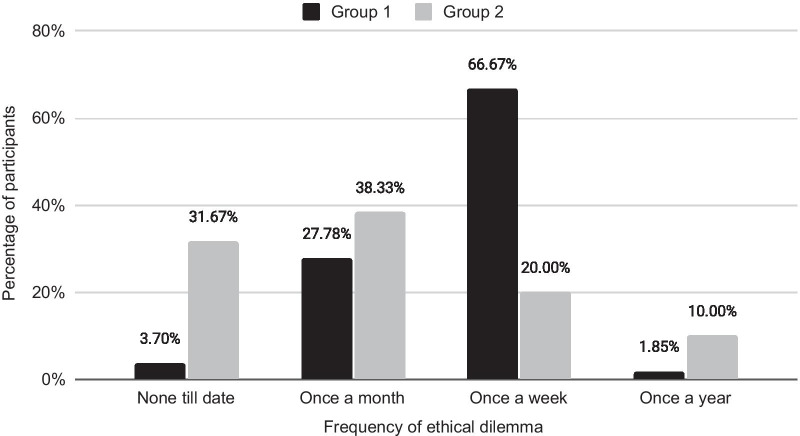
Frequency of ethical issues faced by participants in study 2 (Group 1 n = 54, Group 2 n = 60)

More than half of the respondents in Group 1 (36, 66.67%) claimed to have faced an ethical dilemma at least once a week, while the majority of participants in Group 2 (38.33%, 23) faced it once a month (Fig. 1). Likewise, 31.67% (19) participants in Group 2 claimed to have never encountered an ethical dilemma, while only 3.70% (2) in Group 1 claimed so (Fig. 1). All respondents in both groups thought that medical ethics should be a part of medical education, whereas 96.30% (52) in Group 1 and 98.33% (59) in Group 2 agreed to attend future seminars/workshops on medical ethics if they had the opportunity.

More participants in Group 1 were aware of the Hippocratic Oath and NMC code of ethics compared to Group 2, whereas awareness about the Declaration of Helsinki Oath was low in both groups (see Additional file 1). Only 18.52% (10) respondents from Group 1 were aware of the absence of a clinical ethics committee at PAHS, while 48.33% (29) of the respondents from Group 2 knew about it (see Additional file 1). Among those in Group 1, 94.59% (35) of the participants who had graduated from PAHS itself were unaware of it. Nevertheless, 75.93% (41) respondents in Group 1 and 53.33% (32) respondents in Group 2 knew about the presence of a legal advisor in PAHS. In both the groups (Group 1: median 8 ± IQR 2 and Group 2: median 8 ± IQR 23), respondents have rated the importance of a clinical ethics committee high on a scale from ‘1’ low to ‘10’ high.

Only 11.67% (7) participants in Group 2 considered patients themselves capable of judging what is best for them, while half the participants (28, 51.85%) in Group 1 thought so (see Additional file 1). Participants of Group 1 rated their knowledge of medical ethics higher (median 7 ± IQR 1) compared to those in Group 2 (median 5 ± IQR 3) on a scale from ‘1’ low to ‘10’ high.

### Assessment of knowledge, attitude, and reported practice

Statistically significant difference was found in knowledge (*p* < 0.001), attitude (*p* = 0.001), practice (*p* < 0.001), and overall KAP score (*p* < 0.001) among participants of Group 1 and 2, where Group 1 scored higher (Table 6). Although 14.91% (17) participants claimed to have used resources to complete the questionnaire, no significant difference (*p* = 0.845) was observed in the knowledge score between those who had and had not used resources.

**Table 6:** Assessment of knowledge, practice, attitude and combined KAP of medical ethics for study 2

	N	Measure of central tendency ± dispersion	*p* value
*Knowledge Score (total questions = 22)*			
Group 1	54	14.00 ± 4.00^b^	< 0.001^c^
Group 2	60	12.00 ± 4.00^b^	
*Attitude Score (total questions = 10)*			
Group 1	54	8.00 ± 2.00^b^	0.001^c^
Group 2	60	6.00 ± 2.00^b^	
*Practice Score (total questions = 10)*			
Group 1	54	7.00 ± 3.00^b^	< 0.001^c^
Group 2	60	5.00 ± 2.00^b^	
*KAP Score (total questions = 42)*			
Group 1	54	28.31 ± 5.39^a^	< 0.001^d^
Group 2	60	23.31 ± 4.07^a^	

*N* number of participants

^a^Mean ± SD

^b^Median ± IQR

^c^Mann–Whitney U

^d^Independent T-test

Table 7 describes the performance of study participants in each ethical case scenario. In all nine out of 10 case scenarios presented, more participants from Group 1 scored right in regards to reported practice. The case scenarios in which less than half the participants from Group 1 answered the best practice option were ethical issues arising while treating a minor (Case 4), end-of-life decisions concerning euthanasia (Case 6), and contraception (Case 9) (Table 7). In Group 2, case scenarios where less than half the participants performed well were based on ethical issues on truth-telling (Case 2), treating a minor (Case 4), end-of-life decisions concerning DNR and euthanasia (Case 5, 6), and physician and colleague relationship (Case 10) (Table 7, see Additional file 1).

**Table 7:** Performance of study participants in each case scenario in study 2

Case	Ethical issues related to	Knowledge of presence of breach of ethics n (%)		Knowledge on principle of ethics involved n (%)		Attitude n (%)		Practice n (%)	
		Group 1	Group 2	Group 1	Group 2	Group 1	Group 2	Group 1	Group 2
		(n = 54)	(n = 60)	(n = 54)	(n = 60)	(n = 54)	(n = 60)	(n = 54)	(n = 60)
1	Informed consent	54 (100.00)	51 (85.00)	49 (90.74)	37 (61.67)	50 (92.59)	55 (91.67)	51 (94.44)	51 (85.00)
2	Truth-telling	52 (96.30)	53 (88.33)	42 (77.78)	27 (45.00)	47 (87.04)	41 (68.33)	37 (68.52)	6 (10.00)
3	Confidentiality	43 (79.63)	35 (58.33)	49 (90.74)	50 (83.33)	46 (85.19)	44 (73.33)	46 (85.19)	47 (78.33)
4	Treating minors	30 (55.56)	27 (45.00)	17 (31.48)	17 (28.33)	24 (44.44)	20 (33.33)	23 (42.59)	19 (31.67)
5	End-of-life decisions (DNR)	26 (48.15)	29 (48.33)	28 (51.85)	22 (36.67)	27 (50.00)	26 (43.33)	41 (75.93)	26 (43.33)
6	End-of-life decisions (euthanasia)	45 (83.33)	47 (78.33)	18 (33.33)	27 (45.00)	49 (90.74)	55 (91.67)	24 (44.44)	21 (35.00)
7	End-of-life decisions (withdrawal of treatment)	46 (85.19)	35 (58.33)	41 (75.93)	27 (45.00)	38 (70.37)	23 (38.33)	45 (83.33)	48 (80.00)
8 Part 1	Reportable illness (HIV/AIDS)	29 (53.70)	24 (40.00)	25 (46.30)	12 (20.00)	46 (85.19)	51 (85.00)	38 (70.37)	30 (50.00)
8 Part 2	Reportable illness (HIV/AIDS)	46 (85.19)	56 (93.33)	20 (37.04)	27 (45.00)	-	-	-	-
9	Contraception	23 (42.59)	22 (36.67)	36 (66.67)	18 (30.00)	25 (46.30)	24 (40.00)	23 (42.59)	33 (55.00)
10	Physician and colleague relationship (reporting error)	32 (59.26)	38 (63.33)	25 (46.30)	26 (43.33)	42 (77.78)	47 (78.33)	31 (57.41)	25 (41.67)

In most of the cases, participants were able to correctly identify if there had been a breach of ethics in each of the cases; however, they faced difficulty in identifying the main core principle of ethics involved in treating minors (Case 4), end-of-life decisions related to DNR, euthanasia, withdrawal of treatment (Cases 5, 6, 7), treating patients with HIV/AIDS (Case 8), contraception (Case 9) and physician and colleague relationship (Case 10) (Table 7, see Additional file 1).

## Discussion

Nearly twenty years after most American medical schools had already adopted medical ethics in their curriculum, in 1989, Pellegrino [31] attempted to answer the question: does teaching medical ethics to medical students have a measurable effect on the physicians’ behavior? The author argues that while the evidence in favor of teaching medical ethics was generally lacking, that holds true for other disciplines taught in medical school as well [31]. For example, there is no good evidence, primarily due to the obviousness and, consequently, the lack of studies, that teaching biochemistry, for example, in medical school, improves the quality of a physician’s practice [31]. While that notion might have played a pivotal role in adopting the medical ethics curriculum in medical education in the past, the search for evidence of its relevance continues.

A review of medical ethics education, conducted in 2004, recognized that there are two overlapping views regarding the purpose of teaching medical ethics: (1) to create virtuous physicians and (2) to provide physicians with a skill set for analyzing ethical dilemmas [32]. Whether current ethics teaching–learning methods are fulfilling those objectives or not remains open for discussion [32]. Nevertheless, a consensus regarding the importance of the medical ethics curriculum has been established in the medical community [32–34]. In a study done among deans of medical education and medical ethics course directors at U.S. and Canadian medical schools, 94% of the deans agreed that courses in ethics should be mandatory for all students [35]. A survey of 22 U.K. medical schools enquiring about teaching and assessment in 2006 concluded that medical ethics has an accepted place in the curriculum and should be taught throughout the course [36].

Our study 1 showed that the majority of participants rated the relevance of medical ethics to their work practice high (median 8 ± IQR 3 on a 10 point rating scale). A study conducted in a tertiary care teaching hospital in Barbados, a place with socio-cultural similarities to Nepal, revealed similar results, where 100% of the participating doctors felt that medical ethics was essential to their work [27]. All respondents in our study agreed that medical ethics should be a part of medical education. Studies conducted in Barbados [27] and Northern India [37] revealed similar findings, where 100% and 85% of participants respectively agreed that medical ethics should be taught in medical school.

The current status of knowledge, attitude, and practice of medical ethics might provide some insight into the effectiveness of teaching–learning methods implemented. Many studies have drawn attention to significant deficiencies in understanding medical ethics among medical graduates [38, 39]. Studies conducted in India [40], Nepal [41], and Srilanka [42] also revealed similar conclusions that most doctors’ knowledge regarding clinical ethics was inadequate.

Studies have shown that many physicians are unaware of both the code of ethics of historical importance and the existence of an ethics committee in their institution that functions to facilitate the proper conduct of ethics. A study conducted among physicians in Manipur, India, revealed that more than half of the respondents (54%) were unable to recall any of the contents of the Hippocratic Oath [39]. A similar study conducted in Barbados found out that a significantly lesser number of respondents (11%) did not know the contents of the Hippocratic Oath [27]. In our study, the majority of the participants (Study 1: 66, 93% and Study 2 Group 1: 52, 96.30%, Group 2: 50, 83.33%) were aware of the Hippocratic Oath; however, the knowledge of the contents of the oath was not assessed. The study conducted in Barbados also revealed that over 90% of physicians did not know of the Helsinki Declaration [27]. Similar findings were seen in our Study 2, where only 22.22% (12) participants in Group 1 and 18.33% (11) participants in Group 2 had heard of the Declaration of Helsinki, whereas a higher number of participants (33, 46.48%) in Study 1 were aware of the declaration. More training and workshops need to be organized to educate doctors regarding codes of ethics and their practical implications.

In the study done in Barbados, 29% of physicians were unaware of the existence of an ethics committee at the institution [27]. Our study revealed a higher percentage (Study 1: 35, 52.24%, Study 2 Group 1: 44, 81.48%, Group 2: 31, 51.67%) of respondents were unaware of the absence of a clinical ethics committee at PAHS. PAHS lacks a clinical ethics committee; however, there is a research ethics committee present. The clinical ethics committee supports health professionals in managing complex ethical issues arising during patient management while the research ethics committee reviews research proposals and regulates clinical research [43, 44]. Our study found that 94.59% (35) of the participants who had graduated from PAHS itself were unaware of the absence of a clinical ethics committee at PAHS. This finding could be due to participants confusing the research ethics committee with a clinical ethics committee. This highlights the need to clarify the difference between the two committees during medical ethics lectures.

Most of the respondents (47.6%) in a study in Manipur said they would consult a lawyer or the head of the department or the ethics committee when faced with ethical or legal problems [39]. In research conducted in Barbados, the majority of physicians said they would approach the immediate supervisor first [27]. However, in both our studies 1 and 2, the majority (Study 1: 39, 54.9% and Study 2 Group 1: 39, 72.22%, Group 2: 30, 50%) preferred to consult their colleagues first. This finding could be due to the absence of a clinical ethics committee at PAHS. The reason for doctors approaching colleagues and not the seniors or the department heads to resolve an ethical dilemma needs to be further explored. A blame-free environment where junior doctors feel safe and free to ask questions about ethical issues can be a potential asset in improving ethical practice.

Our results showed that the majority considered doctors as the ones capable of judging what is best for the patient. The fact that the patients have autonomy over their health decisions should be integrated into the curriculum. Also, our study 2 showed that those with adequate exposure to medical ethics education were more likely to judge patients capable of decision making (Group 1: 28, 51.85%, Group 2: 7, 11.67%). As noted by Brogen et al. [39] and Chopra et al. [37], the majority were more likely to lean towards revealing a patient’s condition to the close relatives, irrespective of whether or not they sought the patient’s permission. In our questionnaire, Case 2 presented a vignette where the son of a recently diagnosed cancer patient requests the doctor not to reveal the diagnosis to the patient. Most participants preferred counseling the son and revealing the diagnosis to the patient (Study 1: 33, 45.83%, Study 2 Group 1: 13, 24.07%, Group 2: 36, 60%) over the correct option; talk to the patient and ask whether they want to know the diagnosis (Study 1: 22, 30.56%, Study 2 Group 1: 37, 68.52%, Group 2: 6, 10%). This may reflect the cultural values of Nepalese society, where the head of the family is the decision-maker and hence the advocate for the patient. Often doctors have to deal with the decision-maker without a formal health care proxy status rather than the patient himself, which creates a dilemma regarding autonomy. Another case scenario (Case 4) was about an unmarried 15-year-old girl who comes to the hospital seeking advice on contraceptives. Our participants were divided among the options; 1) advice on contraceptive (Study 1: 37, 51.39%, Study 2 Group 1: 23, 42.59%, Group 2: 19, 31.67%) and 2) ask patient to come with parents (Study 1: 29, 40.28%, Study 2 Group 1: 21, 38.89%, Group 2: 31, 51.67%). This finding may reflect an inadequate grasp on ethical issues regarding emancipated minors among the participants.

### Medical ethics education

The best teaching and learning methodologies and processes for instruction and the optimal strategies for assessment are debatable [33, 34]. In 1989, an increase in moral reasoning was found regardless of the format (lectures vs. case study) of teaching medical ethics [45]. Newer studies have also identified simulation-based medical education to be a favored mode of teaching medical ethics [46, 47]. In regards to assessment, a study done at the University of Toronto intended to measure ethical sensitivity, defined as the ability to recognize that a moral issue exists [48]. The study concluded that clinical vignettes help measure ethical sensitivity in medical students [48].

Most medical schools in Nepal have incorporated some form of medical ethics education curriculum. Variation in the content, teaching and learning method, and dedication of time and other resources in ethics education and assessment suggests that a standard regarding the content of the curriculum and pedagogic methods is lacking. However, a study from Nepal found that 50% of the students felt that ethics teaching in Nepal was not adequate [49]. Teaching–learning methods also vary between different countries. Nevertheless, we found no significant difference in our study 1 regarding the KAP of medical ethics among practitioners who completed their MBBS from Nepal compared to abroad (*p* = 0.261). A similar problem is the lack of consensus in content, teaching–learning method, and assessments of medical ethics among medical schools of the U.S. and Canada, which was concluded by a large multicenter study [35]. A step in the right direction in solving the consensus problem can be developing tools for measuring the effects of teaching medical ethics.

Difficulties in testing the impact of medical ethics education are multifold. Multiple attempts to establish the evidence that teaching medical ethics to students produces virtuous physicians and contributes to their future ethical practice and professionalism have been made. In 1981, a study investigated the effect of teaching medical ethics and found a statistically significant increase in moral reasoning of students exposed to the medical ethics course [45]. One study found a significant increase, as measured by Rest’s Defining Issues Test, in the moral reasoning of medical students after an introductory medical ethics course [50].

The review of 100 articles and three books on medical ethics published from 1978 to 2004 points out two significant problems with studies found in the literature [32]. The first is that very few studies attempt to measure outcomes, and even when they do, the educational goals against which the outcomes are to be measured are poorly defined [32]. The second is that the tools used to assess moral reasoning are adopted from populations other than medical professionals in education [32]. A potential solution can be a tool developed in conjunction with the ethical review board of the institute where the tool is to be implemented. Our study attempted to develop the questionnaire with ethical vignettes containing real-life ethical scenarios reported by medical students as a part of their assessments during the clinical year I. Also, our questionnaire has a clearly defined goal of assessing knowledge, attitude, and practice of ethical principles that the PAHS curriculum intended to deliver [24].

A generally accepted view is that moral reasoning develops with experience as the years of practice increase. We found a positive but weak correlation between the number of years of practice and KAP among the participants in Study 1 (r = 0.313, *p* = 0.016). Our study, however, remains equivocal about training and experience being the primary source of knowledge of medical ethics. The majority of the participants in our Study 1 (46, 63.89%) obtained their knowledge through work experience followed by training during MBBS (44, 61.11%).

Our study 1 found that there was a significant difference between participants who had only completed MBBS and those who had completed postgraduate education in regards to knowledge scores (*p* = 0.050), practice scores (*p* < 0.001), and overall combined KAP scores (*p* = 0.011). However, these findings could also be explained by the fact that postgraduate doctors are also involved in teaching clinical ethics to medical students at PAHS. Nevertheless, a study in Manipur also found that senior doctors had better knowledge of medical ethics [39]. They argued that the difference could be due to more experience, attending more CMEs, conferences, and workshops [39]. Contrary to these findings, a study conducted among several physicians in Germany [51] showed that the longer the duration of practice, the more inadequate the opinion physicians had on patients’ capacities to make decisions regarding their illness. However, our study 2 found that those receiving medical ethics education had significantly higher knowledge score (*p* < 0.001), attitude score (*p* = 0.001), practice score (*p* < 0.001), and overall KAP score (*p* < 0.001) compared to those who had not received formal medical ethics education. A study conducted in Brazil also revealed a higher rate of correct answers among undergraduates who attended lectures on medical ethics compared to those who did not [52]. If or not teaching medical ethics accelerates the learning that comes with experience or deepens the understanding remains to be further investigated.

## Limitations

This study has some limitations. First, it was not practically possible to observe the practice of our respondents; hence only self-reported practice was assessed in this study. Second, Cronbach’s alpha for items testing practice and attitude was low. The study should be carried out in a larger population to check the reliability of the questionnaire and modified accordingly. Third, the questionnaire focused chiefly on clinical and medical ethics and ethically challenging cases related to clinical practice. There was no ethical case scenario related to medical research. The only question related to research ethics included in our questionnaire asked the participants if they had heard of the Declaration of Helsinki and asked them to rate their knowledge regarding it. However, as seen from our findings, many were not aware of the Declaration of Helsinki, making it more essential to include such cases and training into practice. Fourth, multiple principles of ethics are applicable while dealing with an ethical case; however, our study only tested whether participants could identify the main principle involved, though it is still debatable which principle of ethics takes precedence over another. Fifth, although awareness of different codes of ethics and oaths were asked, their contents were not tested. Sixth, there could have been selection bias in recruiting the participants. Participants in study 2 were recruited via convenience sampling. Seventh, both studies were conducted in PAHS alone. More multicenter studies are required to form conclusions that are generalizable to the wider Nepalese medical population.

## Conclusions

This study demonstrated that participants who have received medical ethics education have higher knowledge, attitude, and practice scores. Our findings support the need for medical ethics training to be included as part of the core MBBS curriculum. The study found that the majority of the participants prefer discussing ethical dilemmas with their colleagues. Hence, an environment should be created where the seniors or the head of departments can be easily approached, and the concerned doctors are given appropriate advice within a blame-free environment. This would also encourage junior doctors to report unethical behavior from colleagues and discuss their dilemmas. Moreover, the establishment of a clinical ethics﻿ committee within the hospital may be helpful for the doctors to share their ethical dilemmas and get proper advice when necessary. The study shows that most participants are unaware of the Declaration of Helsinki. Research ethics should also, therefore, be included and given priority in medical ethics education. Also, most participants, even those who were graduates of PAHS, were unaware of the absence of a clinical ethics committee at PAHS, prompting the need to explain the importance of a clinical ethics committee and its distinction from a research ethics committee during medical ethics lectures. Participants in this study demonstrated poor reported practice on ethical issues concerning truth-telling, end-of-life decisions, treating HIV/AIDS patients, treating minors, and reporting colleague’s errors. During medical ethics training, such ethical issues should be prioritized and discussed in a country-specific context.

## Supplementary Information

**Additional file 1.** Supplementary Results and Findings.

**Additional file 2.** Informed Consent form and Survey questionnaire for Study 1.

**Additional file 3.** Informed Consent form and Survey questionnaire for Study 2.

## Data Availability

The datasets supporting the conclusions of this article will be made available by the corresponding author on request.

## Abbreviations

BPKIHS: B.P. Koirala Institute of Health Sciences
CBLE: Community Based Learning and Education
ICM: Introduction to Clinical Medicine
IRC: Institutional Review Committee
IQR: Inter-quartile range
KAP: Knowledge attitude and practice
K.U.: Kathmandu University
KUSMS: Kathmandu University School of Medical Sciences
MBBS: Bachelor of Medicine and Bachelor of Surgery
MCQs: Multiple Choice Questions
MD/MS: Doctor of Medicine/Masters of Surgery
NMC: Nepal Medical Council
PAHS: Patan Academy of Health Sciences
PBL: Problem Based Learning
PBQs: Problem Based Questions
SD: Standard deviation
T.U.: Tribhuvan University
WHO: World Health Organization
WMA: World Medical Association

## References

[1] Venes D, editor. Taber’s cyclopedic medical dictionary. 20th ed. Pennsylvania: F.A. Davis Company; 2005.

[2] Williams JR. Medical Ethics Manual [Internet]. Ferney-Voltaire, France: The World Medical Association; 2015. https://www.wma.net/wp-content/uploads/2016/11/Ethics_manual_3rd_Nov2015_en.pdf. Accessed 20 April 2021.

[3] Singer PA, Pellegrino ED, Siegler M (2001). Clinical ethics revisited. BMC Med Ethics.

[4] Nepal Medical Council. Code of Ethics and Professional Conducts [Internet]. Kathmandu, Nepal: Nepal Medical Council; 2017. https://nmc.org.np/files/4/Code%20of%20Ethics_Print_version.pdf. Accessed 25 April 2021.

[5] Raut S, Kumar A (2018). Medical ethics in clinical practice in Nepal: challenges and way forward. J Univ Coll Med Sci.

[6] World Health Organization. Module for Teaching Medical Ethics to Undergraduates [Internet]. India: World Health Organization Regional Office for South-East Asia; 2009. https://apps.who.int/iris/bitstream/handle/10665/205534/B4401.pdf?sequence=1&isAllowed=y. Accessed 11 May 2021.

[7] Imran M, Samad S, Maaz M, Qadeer A, Najmi AK, Aqil M (2013). Hippocratic oath and conversion of ethico-regulatory aspects onto doctors as a physician, private individual and a clinical investigator. J Midlife Health.

[8] Haan MM, van Gurp JLP, Naber SM, Groenewoud AS (2018). Impact of moral case deliberation in healthcare settings: a literature review. BMC Med Ethics.

[9] Steinkamp N, Gordijn B (2003). Ethical case deliberation on the ward. A comparison of four methods. Med Health Care Philos.

[10] Amnesty International. Ethical codes and declarations relevant to the health professions. 4th ed. London: Amnesty International Publications; 2000.

[11] Summers J, Morrison E. Principles of healthcare ethics. In: Health care ethics. 2nd ed. Sudbury: Jones and Bartlett Publishers; 2009. p. 41–58.

[12] Hafferty FW, Franks R (1994). The hidden curriculum, ethics teaching, and the structure of medical education. Acad Med.

[13] Kavas MV, Ulman YI, Demir F, Artvinli F, Şahiner M, Demirören M (2020). The state of ethics education at medical schools in Turkey: taking stock and looking forward. BMC Med Educ.

[14] Miles SH, Lane LW, Bickel J, Walker RM, Cassel CK (1989). Medical ethics education: coming of age. Acad Med.

[15] The World Medical Association. WMA Resolution on the Inclusion of Medical Ethics and Human Rights in the Curriculum of Medical Schools World-Wide [Internet]. World Medical Association; 2021. https://www.wma.net/policies-post/wma-resolution-on-the-inclusion-of-medical-ethics-and-human-rights-in-the-curriculum-of-medical-schools-world-wide/. Accessed 16 May 2021.

[16] Miyasaka M, Akabayashi A, Kai I, Ohi G (1999). An international survey of medical ethics curricula in Asia. J Med Ethics.

[17] Kasturiaratch N, Lie R, Seeberg J. Health ethics in South-East Asia, Vol I : Health ethics in six SEAR countries [Internet]. New Delhi: WHO Regional Office for South-East Asia; 1999. https://apps.who.int/iris/handle/10665/205216. Accessed 18 May 2021.

[18] Adhikari RK (2013). Ethics in undergraduate medical courses in Nepal. Kathmandu Univ Med J.

[19] Dixit H (2003). Role of Nepal medical council in MBBS curriculum. Kathmandu Univ Med J.

[20] Tribhuvan University Institute of Medicine. Curriculum for Bachelor of Medicine and Bachelor of Surgery. Maharajgunj, Kathmandu: Medical Education Department; 2008.

[21] Kathmandu University School of Medical Sciences (2006). MBBS Curriculum Clinical Sciences.

[22] BPKIHS. The MBBS Curriculum of B.P. Koirala Institute of Health Sciences. 2nd ed. Dharan: B.P. Koirala Institute of Health Sciences; 2014.

[23] Shrestha S (2020). Development and implementation of clinical presentation curriculum at PAHS School of Medicine. J Patan Acad Health Sci.

[24] Patan Academy of Health Sciences (2010). Curriculum for MBBS program of Patan Academy of Health Sciences.

[25] Baral K, Allison J, Upadhyay S, Bhandary S, Shrestha S, Renouf T. Rural community as context and teacher for health professions education. Cureus. 2016;8(11):e866. 10.7759/cureus.866 PMC5143305 27980887

[26] Nepal Medical Council. National Guidelines for Medical Internship Training [Internet]. Kathmandu, Nepal: Nepal Medical Council; 2007. https://nmc.org.np/files/4/Internship%20Guideline.pdf. Accessed 20 April 2021.

[27] Hariharan S, Jonnalagadda R, Walrond E, Moseley H (2006). Knowledge, attitudes and practice of healthcare ethics and law among doctors and nurses in Barbados. BMC Med Ethics.

[28] Toy E, Raine S, Cochrane T (2015). Case files medical ethics and professionalism.

[29] Fisher C (2006). Kaplan medical USMLE medical ethics: the 100 cases you are most likely to see on the test.

[30] Spandorfer J, Pohl CA, Rattner SL, Nasca TJ (2009). Professionalism in medicine: a case-based guide for medical students.

[31] Pellegrino ED (1989). Teaching medical ethics: some persistent questions and some responses. Acad Med.

[32] Eckles RE, Meslin EM, Gaffney M, Helft PR (2005). Medical ethics education: where are we? Where should we be going? A review. Acad Med.

[33] Persad GC, Elder L, Sedig L, Flores L, Emanuel EJ (2008). The current state of medical school education in bioethics, health law, and health economics. J Law Med Ethics.

[34] Lakhan SE, Hamlat E, McNamee T, Laird C (2009). Time for a unified approach to medical ethics. Philos Ethics Humanit Med.

[35] Lehmann LS, Kasoff WS, Koch P, Federman DD (2004). A survey of medical ethics education at U.S. and Canadian medical schools. Acad Med.

[36] Mattick K, Bligh J (2006). Teaching and assessing medical ethics: where are we now?. J Med Ethics.

[37] Chopra M, Bhardwaj A, Mithra P, Singh A, Siddiqui A, Dr R (2013). Current status of knowledge, attitudes and practices towards healthcare ethics among doctors and nurses from Northern India—A Multicentre Study. J Krishna Inst Med Sci Univ.

[38] Arun Babu T, Venkatesh C, Sharmila V (2013). Are tomorrow’s doctors aware of the code of medical ethics?. Indian J Med Ethics.

[39] Brogen AS, Rajkumari B, Laishram J, Joy A (2009). Knowledge and attitudes of doctors on medical ethics in a teaching hospital, Manipur. Indian J Med Ethics.

[40] Subramanian T, Mathai AK, Kumar N (2013). Knowledge and practice of clinical ethics among healthcare providers in a government hospital, Chennai. Indian J Med Ethics.

[41] Adhikari S, Paudel K, Aro AR, Adhikari TB, Adhikari B, Mishra SR (2016). Knowledge, attitude and practice of healthcare ethics among resident doctors and ward nurses from a resource poor setting, Nepal. BMC Med Ethics.

[42] Ranasinghe AWIP, Fernando B, Sumathipala A, Gunathunga W (2020). Medical ethics: knowledge, attitude and practice among doctors in three teaching hospitals in Sri Lanka. BMC Med Ethics.

[43] Crico C, Sanchini V, Casali PG, Pravettoni G (2021). Evaluating the effectiveness of clinical ethics committees: a systematic review. Med Health Care Philos.

[44] Hemminki E (2016). Research ethics committees in the regulation of clinical research: comparison of Finland to England, Canada, and the United States. Health Res Policy Syst.

[45] Self DJ, Wolinsky FD, Baldwin DC (1989). The effect of teaching medical ethics on medical students’ moral reasoning. Acad Med.

[46] Henderson H, Ballard I, Alsuwaidi L, Thomas R, Ezimokhai M (2018). Simulation: teaching medical ethics to first year medical students within the United Arab Emirates. MedEdPublish.

[47] AlMahmoud T, Hashim MJ, Elzubeir MA, Branicki F (2017). Ethics teaching in a medical education environment: preferences for diversity of learning and assessment methods. Med Educ Online.

[48] Hébert PC, Meslin EM, Dunn EV (1992). Measuring the ethical sensitivity of medical students: a study at the University of Toronto. J Med Ethics.

[49] Marahatta SB, Dixit H (2008). Students’ perception regarding medical education in Nepal. Kathmandu Univ Med J.

[50] Self DJ, Baldwin DC, Wolinsky FD (1992). Evaluation of teaching medical ethics by an assessment of moral reasoning. Med Educ.

[51] Wandrowski J, Schuster T, Strube W, Steger F (2012). Medical ethical knowledge and moral attitudes among physicians in Bavaria. Dtsch Arztebl Int.

[52] das Graças VBA, de Souza JF, Santos JGMS, Almeida MFA, Oliveira EVG, Santos NVMO (2019). Knowledge about medical ethics and conflict resolution during undergraduate courses. Rev Bioét.

