# Supplementary material for: Does teaching medical ethics ensure good knowledge, attitude, and reported practice? An ethical vignette-based cross-sectional survey among doctors in a tertiary teaching hospital in Nepal
Source: BMC Med Ethics. 2021 Aug 5;22:109. doi: 10.1186/s12910-021-00676-6 (PMC8340509; doi:10.1186/s12910-021-00676-6)
Supplement: Supplementary file 1 — Additional file 1. Supplementary Results and Findings. [file 12910_2021_676_MOESM1_ESM.docx]

| **Table A Participant's reported practice in different case scenarios in Study 1** |  |  |
| --- | --- | --- |
|  |  |  |
| **Informed Consent (N = 72)** | **n** | **%** |
| **Case Scenario 1 A doctor needs to perform a lumbar puncture on a fully conscious man** | | |
| Ask your junior intern to take informed consent from the patient | 1 | 1.39 |
| Take informed consent from the wife alone after explaining to her the risks and benefits of undergoing LP | 2 | 2.78 |
| Take informed consent from the patient after explaining him the risks and benefits of undergoing LP | 69 | 95.83 |
| Do lumbar puncture without taking consent from anyone | 0 | 0.00 |
|  |  |  |
| **Truth-telling (N = 72)** |  |  |
| **Case Scenario 2 A old man is diagnosed of cancer and his son asks the doctor not to tell the diagnosis to his father** | | |
| Talk with the patient if he wants to know about the diagnosis | 22 | 30.56 |
| Counsel the son and tell the patient about his diagnosis | 33 | 45.83 |
| Inform close relatives and come to a consensus on whether to tell the patient about his diagnosis or not | 13 | 18.06 |
| Not tell the patient about his diagnosis respecting the son’s request | 4 | 5.56 |
|  |  |  |
| **Confidentiality (N = 72)** |  |  |
| **Case Scenario 3 A conscious man is hospitalized with chest pain and his alleged wife asks the doctor to tell her about his condition** | | |
| Discuss with your senior | 3 | 4.17 |
| Talk to the patient if he wants to tell his wife | 61 | 84.72 |
| Give the wife details about the patient without asking the patient | 7 | 9.72 |
| Ignore the wife’s demands | 1 | 1.39 |
|  |  |  |
| **Treating minors (N = 72)** |  |  |
| **Case 4 A 15-year-old unmarried girl comes alone and asks the doctor advice on the use of contraceptives** | | |
| Advise the patient regarding use of contraceptive | 37 | 51.39 |
| Refuse to give advice on grounds of underage | 4 | 5.56 |
| Advise her to come back with her parents | 29 | 40.28 |
| Consult with your senior | 2 | 2.78 |
|  |  |  |
| **End-of-life decisions (DNR) (N = 72)** |  |  |
| **Case Scenario 5 A 64 year old man with a DNR on the grounds of RCC presents after a fall and is diagnosed to have fracture of neck of femur. The patient’s family wants him to undergo surgery.** | | |
| Discuss with your senior | 7 | 9.72 |
| Ask the patient if he wants to undergo surgery for neck of femur fracture | 49 | 68.06 |
| Counsel the patient’s family that since he is DNR, no intervention can be done on him | 16 | 22.22 |
| Undergo surgery for neck of femur fracture without consent from anyone | 0 | 0.00 |
|  |  |  |
| **End-of-life decisions (Euthanasia) (N = 72)** |  |  |
| **Case Scenario 6 Parents want the doctor to terminate the life of a baby born with hydrocephalus with poor outcome.** | | |
| Tell the parents that terminating life is illegal in Nepal and counsel child might survive | 31 | 43.06 |
| Medically terminate the child | 6 | 8.33 |
| Ignore the parent’s requests and put the child on ventilator | 0 | 0.00 |
| Advise the parent that the child might survive and that the best option would be to discontinue active intensive care and continue with normal care | 35 | 48.61 |
|  |  |  |
| **End-of-life decisions (Withdrawal of treatment) (N = 71)** |  |  |
| **Case Scenario 7 A 60 year old patient with diabetic nephropathy, no depression asks the doctor to discontinue dialysis.** | | |
| Make him sign an informed consent and discontinue dialysis | 2 | 2.78 |
| Tell his family members about his decision | 0 | 0.00 |
| Simply discontinue the treatment | 14 | 19.44 |
| Explain the consequences, get an informed consent form signed, and then discontinue dialysis | 55 | 76.39 |
|  |  |  |
| **Reportable illness (HIV/AIDS) (N = 72)** |  |  |
| **Case Scenario 8 A 10 weeks pregnant woman tests positive for HIV and asks the doctor not to tell the diagnosis to her husband.** | | |
| Counsel the patient on telling her husband about her HIV status and tell her husband even if she is unwilling | 34 | 47.22 |
| Tell her husband about his wife's HIV positive status and ask him to undergo HIV test | 14 | 19.44 |
| Counsel the patient on telling her husband and not tell the husband if she is unwilling | 24 | 33.33 |
| Not tell the husband | 0 | 0.00 |
|  |  |  |
| **Contraception (N = 72)** |  |  |
| **Case Scenario 9 A G4P3 lady is about to undergo caesarean section. She wants permanent sterilization but her husband disagrees.** | | |
| Inform the husband without the patient’s knowledge and try to counsel him | 0 | 0.00 |
| Perform the operation | 4 | 5.56 |
| Tell the patient to discuss about it with her husband first but agree to perform the surgery anyway | 40 | 55.56 |
| Do not perform the operation without the husband's consent | 28 | 38.89 |
|  |  |  |
| **Physician and colleague relationship (Reporting error) (N = 72)** |  |  |
| **Case Scenario 10 A colleague fails to inquire about LMP and perform a UPT in a sexually active woman presenting with abdominal pain. A day later she is diagnosed with ruptured ectopic pregnancy.** | | |
| Directly report the error of the previous doctor to the Nepal Medical Council | 1 | 1.39 |
| Inform the previous doctor about his error and also report the error to the Department Chair | 28 | 38.89 |
| Avoid telling the patient, the previous doctor or the Department Chair about the previous doctor’s error | 6 | 8.33 |
| Inform the previous doctor of his error and advice him not to repeat such mistakes | 37 | 51.39 |

| **Table B Participant's reported practice in different case scenarios in Study 2** | | | | |
| --- | --- | --- | --- | --- |
|  |  |  |  |  |
|  | **Group 1 (n = 54)** | | **Group 2 (n = 60)** | |
| **Informed Consent** | **n** | **%** | **n** | **%** |
| **Case Scenario 1 A doctor needs to perform a lumbar puncture on a fully conscious man** | | | | |
| Ask your junior intern to take informed consent from the patient | 2 | 3.70 | 0 | 0.00 |
| Take informed consent from the wife alone after explaining to her the risks and benefits of undergoing LP | 1 | 1.85 | 9 | 15.00 |
| Take informed consent from the patient after explaining him the risks and benefits of undergoing LP | 51 | 94.44 | 51 | 85.00 |
| Do lumbar puncture without taking consent from anyone | 0 | 0.00 | 0 | 0.00 |
|  |  |  |  |  |
| **Truth-telling** |  |  |  |  |
| **Case Scenario 2 A old man is diagnosed of cancer and his son asks the doctor not to tell the diagnosis to his father** | | | | |
| Talk with the patient if he wants to know about the diagnosis | 37 | 68.52 | 6 | 10.00 |
| Counsel the son and tell the patient about his diagnosis | 13 | 24.07 | 36 | 60.00 |
| Inform close relatives and come to a consensus on whether to tell the patient about his diagnosis or not | 2 | 3.70 | 9 | 15.00 |
| Not tell the patient about his diagnosis respecting the son’s request | 2 | 3.70 | 9 | 15.00 |
|  |  |  |  |  |
| **Confidentiality** |  |  |  |  |
| **Case Scenario 3 A conscious man is hospitalized with chest pain and his alleged wife asks the doctor to tell her about his condition** | | | | |
| Discuss with your senior | 4 | 7.41 | 2 | 3.33 |
| Talk to the patient if he wants to tell his wife | 46 | 85.19 | 47 | 78.33 |
| Give the wife details about the patient without asking the patient | 3 | 5.56 | 11 | 18.33 |
| Ignore the wife’s demands | 1 | 1.85 | 0 | 0.00 |
|  |  |  |  |  |
| **Treating minors** |  |  |  |  |
| **Case 4 A 15-year-old unmarried girl comes alone and asks the doctor advice on the use of contraceptives** | | | | |
| Advise the patient regarding use of contraceptive | 23 | 42.59 | 19 | 31.67 |
| Refuse to give advice on grounds of underage | 4 | 7.41 | 6 | 10.00 |
| Advise her to come back with her parents | 21 | 38.89 | 31 | 51.67 |
| Consult with your senior | 6 | 11.11 | 4 | 6.67 |
|  |  |  |  |  |
| **End-of-life decisions (DNR)** |  |  |  |  |
| **Case Scenario 5 A 64 year old man with a DNR on the grounds of RCC presents after a fall and is diagnosed to have fracture of neck of femur. The patient’s family wants him to undergo surgery.** | | | | |
| Discuss with your senior | 6 | 11.11 | 14 | 23.33 |
| Ask the patient if he wants to undergo surgery for neck of femur fracture | 41 | 75.93 | 26 | 43.33 |
| Counsel the patient’s family that since he is DNR, no intervention can be done on him | 7 | 12.96 | 20 | 33.33 |
| Undergo surgery for neck of femur fracture without consent from anyone | 0 | 0.00 | 0 | 0.00 |
|  |  |  |  |  |
| **End-of-life decisions (Euthanasia)** |  |  |  |  |
| **Case Scenario 6 Parents want the doctor to terminate the life of a baby born with hydrocephalus with poor outcome.** | | | | |
| Tell the parents that terminating life is illegal in Nepal and counsel child might survive | 27 | 50.00 | 38 | 63.33 |
| Medically terminate the child | 3 | 5.56 | 1 | 1.67 |
| Ignore the parent’s requests and put the child on ventilator | 0 | 0.00 | 0 | 0.00 |
| Advise the parent that the child might survive and that the best option would be to discontinue active intensive care and continue with normal care | 24 | 44.44 | 21 | 35.00 |
|  |  |  |  |  |
| **End-of-life decisions (Withdrawal of treatment)** |  |  |  |  |
| **Case Scenario 7 A 60 year old patient with diabetic nephropathy, no depression asks the doctor to discontinue dialysis.** | | | | |
| Make him sign an informed consent and discontinue dialysis | 6 | 11.11 | 0 | 0.00 |
| Tell his family members about his decision | 0 | 0.00 | 0 | 0.00 |
| Simply discontinue the treatment | 3 | 5.56 | 12 | 20.00 |
| Explain the consequences, get an informed consent form signed, and then discontinue dialysis | 45 | 83.33 | 48 | 80.00 |
|  |  |  |  |  |
| **Reportable illness (HIV/AIDS)** |  |  |  |  |
| **Case Scenario 8 A 10 weeks pregnant woman tests positive for HIV and asks the doctor not to tell the diagnosis to her husband.** | | | | |
| Counsel the patient on telling her husband about her HIV status and tell her husband even if she is unwilling | 38 | 70.37 | 30 | 50.00 |
| Tell her husband about his wife's HIV positive status and ask him to undergo HIV test | 6 | 11.11 | 20 | 33.33 |
| Counsel the patient on telling her husband and not tell the husband if she is unwilling | 10 | 18.52 | 10 | 16.67 |
| Not tell the husband | 0 | 0.00 | 0 | 0.00 |
|  |  |  |  |  |
| **Contraception** |  |  |  |  |
| **Case Scenario 9 A G4P3 lady is about to undergo caesarean section. She wants permanent sterilization but her husband disagrees.** | | | | |
| Inform the husband without the patient’s knowledge and try to counsel him | 0 | 0.00 | 0 | 0.00 |
| Perform the operation | 4 | 7.41 | 4 | 6.67 |
| Tell the patient to discuss about it with her husband first but agree to perform the surgery anyway | 23 | 42.59 | 33 | 55.00 |
| Do not perform the operation without the husband's consent | 27 | 50.00 | 23 | 38.33 |
|  |  |  |  |  |
| **Physician and colleague relationship (Reporting error)** |  |  |  |  |
| **Case Scenario 10 A colleague fails to inquire about LMP and perform a UPT in a sexually active woman presenting with abdominal pain. A day later she is diagnosed with ruptured ectopic pregnancy.** | | | | |
| Directly report the error of the previous doctor to the Nepal Medical Council | 0 | 0.00 | 0 | 0.00 |
| Inform the previous doctor about his error and also report the error to the Department Chair | 31 | 57.41 | 25 | 41.67 |
| Avoid telling the patient, the previous doctor or the Department Chair about the previous doctor’s error | 4 | 7.41 | 3 | 5.00 |
| Inform the previous doctor of his error and advice him not to repeat such mistakes | 19 | 35.19 | 32 | 53.33 |

| **Table C Participant's assessment of who they thought was best capable of judging what is best for the patient in Study 1 (N = 71)** | | | |
| --- | --- | --- | --- |
|  |  |  |  |
| **Judge** | **n (%)** |  |  |
| Doctor | 50 (70.42) |  |  |
| Patient themselves | 21 (29.58) |  |  |
|  |  |  |  |
| **Table D Participant's assessment of who they thought was best capable of judging what is best for the patient in Study 1 (N = 71)** | | | |
|  |  |  |  |
|  | **Group 1 (n = 54)** | **Group 2 (n = 60)** |  |
| **Judge** | **n (%)** | **n (%)** |  |
| Doctor | 23 (42.59) | 48 (80.00) |  |
| Patient themselves | 28 (51.85) | 7 (11.67) |  |
| Others | 3 (5.56) | 5 (8.33) |  |
| Note: Others included patient relatives, legal advisor | | |  |

| **Table E Participant preference on who to consult when an ethical dilemma arises in study 1 (N = 71)** | | | | |
| --- | --- | --- | --- | --- |
|  | **n** | **%** |  |  |
| Head of Department | 32 | 45.10 |  |  |
| Colleagues | 39 | 54.90 |  |  |

| **Table F Participant preference on who to consult when an ethical dilemma arises in study 2** | | | | |
| --- | --- | --- | --- | --- |
|  | **Group 1 (n = 54)** | | **Group 2 (n = 60)** | |
|  | **n** | **%** | **n** | **%** |
| Colleagues | 39 | 72.22 | 30 | 50.00 |
| Head of the department | 11 | 20.37 | 23 | 38.33 |
| Others | 4 | 7.41 | 7 | 11.67 |
| Note: Others include legal advisor, hospital administrator, supervisors | | | | |

| **Table F Participant knowledge of if an ethics committee is present at PAHS in study 1 (N = 67)** | | | | | |
| --- | --- | --- | --- | --- | --- |
|  |  |  |  |  |  |
|  | **n** | **%** |  |  |  |
| Right | 32 | 47.76 |  |  |  |
| Wrong | 35 | 52.24 |  |  |  |

| **Table G Participant knowledge of if an ethics committee is present at PAHS in study 2** | | | | |
| --- | --- | --- | --- | --- |
|  | **Group 1 (n = 54)** | | **Group 2 (n = 60)** | |
|  | **n** | **%** | **n** | **%** |
| Right | 10 | 18.52 | 29 | 48.33 |
| Wrong | 44 | 81.48 | 31 | 51.67 |

**Table H Source of knowledge of medical ethics for study 2**

|  | **Group 1 (n = 54)** | **Group 2 (n = 60)** |
| --- | --- | --- |
|  | **n (%)** | **n (%)** |
| Work Experience | 39 (72.22) | 30 (50.00) |
| Lectures During MBBS | 50 (92.59) | 42 (70.00) |
| Books/Literature | 28 (51.85) | 22 (36.67) |
| Seminar/Workshops/CME | 15 (27.78) | 11 (18.33) |

| **Table I Participants awareness of code of medical ethics in study 2** |  |  |
| --- | --- | --- |
| **Code of Ethics** | **Group 1 (n = 54)** | **Group 2 (n = 60)** |
|  | **n (%)** | **n (%)** |
| Hippocratic Oath | 52 (96.30) | 50 (83.33) |
| Nepal Medical Council Code of Ethics | 52 (96.30) | 45 (75.00) |
| Declaration of Helsinki | 12 (22.22) | 11 (18.33) |

| **Table J Correlation between years of practice after MBBS and KAP score in Study 1** | | | |
| --- | --- | --- | --- |
|  | **N** | **Rho (Correlation Coefficient)** | **p-value** |
| Knowledge score | 62 | 0.218 | 0.088⁺ |
| Attitude score | 66 | 0.262 | 0.034⁺ |
| Practice score | 67 | 0.476 | <0.001⁺ |
| Combined KAP score | 59 | 0.313 | 0.016⁺ |
| **⁺ Spearman's** |  |  |  |

**
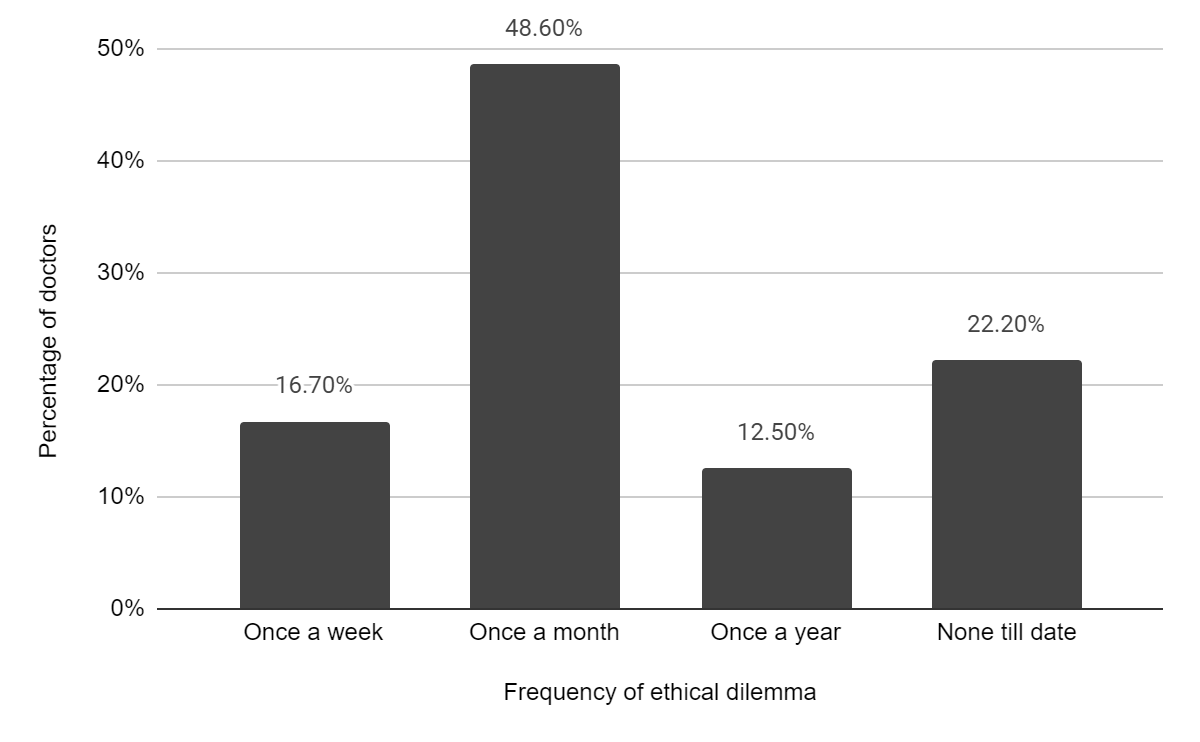
**

**Figure A Frequency of Ethical Dilemmas Faced by Clinicians at PAHS in study 1 (n = 72)**
