# Supplementary material for: Does teaching medical ethics ensure good knowledge, attitude, and reported practice? An ethical vignette-based cross-sectional survey among doctors in a tertiary teaching hospital in Nepal
Source: BMC Med Ethics. 2021 Aug 5;22:109. doi: 10.1186/s12910-021-00676-6 (PMC8340509; doi:10.1186/s12910-021-00676-6)
Supplement: Supplementary file 2 — Additional file 2. Informed Consent form and Survey questionnaire for Study 1. [file 12910_2021_676_MOESM2_ESM.pdf]

IRC-PAHS

Institutional Review Committee - Patan Academy of Health Sciences

पाटन स्वास्थ्य विज्ञान प्रतिष्ठान

Lagankhel-5, Lalitpur, Nepal लगनखेल- ५, ललितपुर, नेपाल

Chairperson

Prof. Dr. Jay N Shah

Member secretary

Assoc. Prof. Dr. Nabees MS Pradhan

Members

Assoc. Prof. Dr. Alka Singh

Prof. Shiv Ram Prasad Koirala

Asst. Prof. Ira Shrestha

Sr. Rachana Shakya

Asst. Prof . Shital Bhandary

Lecturer Dr. Vivek K. Todi

Lecturer Dr. Ashis Shrestha

Ref: std1508031078
Date: 2015-08-03

To:
Ashma Shrestha,
PAHS-MBBS Batch 2
Group 5

Thank you for submission of your research proposal. This is to inform that your proposal has been approved by “IRC-PAHS”. We are confident that you will follow the guidelines, and provide necessary information/materials as and when required by the “IRC-PAHS”. For any queries please contact IRC-PAHS secretary/chairman.

Title of study: “Knowledge, Attitude and Practice of Medical Ethics among Clinicians at Patan Academy of Health Sciences.”

Principle Investigator: Ashma Shrestha, Medical Student, Patan Academy of Health Sciences (PAHS), Kathmandu, Nepal.

Sincerely,

Assoc. Prof. Dr. Nabees Man Singh Pradhan
Member Secretary, IRC-PAHS
Patan Academy of Health Sciences (PAHS), Ktm, Nepal
Phone:977-1-5521034, Fax: 977-1-5548008, Cell: 977-1-9851094037
irc-pahs@pahs.edu.np,nabeesman@gmail.com
