# Supplementary material for: Does teaching medical ethics ensure good knowledge, attitude, and reported practice? An ethical vignette-based cross-sectional survey among doctors in a tertiary teaching hospital in Nepal
Source: BMC Med Ethics. 2021 Aug 5;22:109. doi: 10.1186/s12910-021-00676-6 (PMC8340509; doi:10.1186/s12910-021-00676-6)
Supplement: Supplementary file 3 — Additional file 3. Informed Consent form and Survey questionnaire for Study 2. [file 12910_2021_676_MOESM3_ESM.pdf]

पाटन स्वास्थ्य विज्ञान प्रतिष्ठान
Patan Academy of Health Sciences
(पाटन स्वास्थ्य विज्ञान प्रतिष्ठान ऐन, २०६४ अन्तर्गत स्थापित)
लगनखेल-५, ललितपुर, नेपाल Lagankhel-5, Lalitpur,Nepal

Date: March 14, 2017

To:

Carmina Shrestha

PAHS-MBBS Batch 2

Thank you for submission of your proposal for **Quality Improvement Project** as part of elective posting during internship program. This is to inform that your proposal has been approved by the elective committee at Patan Academy of Health Sciences. We are confident that you will follow the appropriate guidelines for electronic data collection. For any queries, please contact the elective committee secretary.

Title of study: "Comparison of knowledge, attitude and practice of medical ethics among medical officers with and without formal medical ethics training"

Principal Investigator: Carmina Shrestha, Intern, Patan Academy of Health Sciences (PAHS), Kathmandu, Nepal

Sincerely,

Dr. Rabi Shakya

Chair, Elective Committee – PAHS

Phone:977-1-5522266

Email: electivecommittee@pahs.edu.np

E-mail: pahs@pahs.edu.np Tel: +977-1-5545112 Fax: +977-1-5545114 G.P.O. Box 26500
